# An emotional support model in aging families: linking interaction patterns to positive mental health through social activity engagement and aging attitudes

**DOI:** 10.3389/fpubh.2025.1531110

**Published:** 2025-04-08

**Authors:** Jianqian Wu, Yixuan Li, Qiuling Chao

**Affiliations:** ^1^School of Public Policy and Administration, Xi'an Jiaotong University, Xi'an, Shaanxi, China; ^2^School of New Media Art, Xi'an Polytechnic University, Xi'an, Shaanxi, China

**Keywords:** positive mental health, family emotional interaction, attitudes toward aging, social activity engagement, older adults

## Abstract

**Objectives:**

Family emotional interactions can be both supportive and detrimental, and their impact on positive mental health remains unclear. The study aimed to investigate the association and underlying mechanisms between family emotional interaction and positive mental health.

**Methods:**

A cross-sectional study was conducted among 1,200 older adults from Shanxi, Shaanxi, and Henan provinces in China. Data were collected via a questionnaire package assessing older adults' positive mental health, family emotional interaction, social activity engagement, and attitudes toward aging. Statistical analyses were performed using descriptive statistics, Pearson correlation, and path analysis.

**Results:**

Social activity engagement, psychological growth, physiological change, and emotional support were positively associated with positive mental health, and negative interaction was negatively associated with positive mental health. Emotional support was the main effect of promoting positive mental health among older adults. Social activity engagement and positive attitudes toward aging mediated the association between emotional support and positive mental health.

**Conclusion:**

This study presented an emotional support model among older adults. Being emotionally supported by families and actively engaging in social activities positively affects older adults' self-perceptions and promotes positive mental health, which are essential strategies for older adults to cope with potential stressors in later life. These findings advocate integrating family-centered interventions within gerontological practice to optimize emotional support across aging trajectories.

## 1 Introduction

Family interaction refers to face-to-face communication between older adults and their families, including spouses and adult children. Distinguishing between instrumental and informational interactions, family emotional interaction provides a vital source of emotional support for older adults. It significantly affects older adults' ability to maintain cognitive function, health, and wellbeing (1). However, family emotional interactions are not always supportive; they are also detrimental. Providing emotional support to older adults is crucial to aging services. In this study, we first revise a family emotional interaction questionnaire for older adults to distinguish and measure supportive and negative family emotional interactions. We then further examine the association and underlying mechanisms between family emotional interaction and positive mental health by constructing an emotional support model.

### 1.1 Theoretical background

The social convoy model identified older adults' interaction networks and interaction quality (2). Spouses and adult children are central to older adults' social networks. They are generally the people older adults interact with in the most consistent, frequent, and influential way. High-quality family interaction means a good match between one's needs and the interactions, with positive and pleasant experiences throughout the interaction (3). On the contrary, if older adults receive mostly negative emotional interactions, such as arguments, indifference, and neglect, they will always be stressed and prone to illness or even lose their willingness to live (4, 5).

Investigating older adults' family emotional interactions is one primary focus of our study. This study draws on Lincoln et al.'s (6) research to conceptualize family emotional interactions into two dimensions: emotional support and negative interactions. Emotional support is a practical resource delivered through interpersonal interactions designed to meet an individual's emotional and relational belongingness needs (e.g., support, encouragement, and caring). Negative interactions are verbal/non-verbal behaviors that can diminish an individual's sense of self-worth and hurt feelings (e.g., devaluation, ridicule, and neglect). Accordingly, the present study revised a family emotional interaction questionnaire for older adults.

### 1.2 Literature review

Existing research has focused on the relationship between social interactions and older adults' cognitive and physical health. However, the association between family emotional interaction and positive mental health among older adults remains unexplored. Social interaction could enhance cognitive performance among older adults by activating and maintaining the efficiency of brain networks. A higher quality of social interaction is accompanied by a 46% decrease in dementia likelihood (7). In contrast, negative social interaction was a high-risk factor for cognitive decline and mild cognitive impairment (8). Negative interaction predicted tremendous perceived stress and high blood pressure (9). Our study argues that similar to the effects of social interaction, family emotional interaction may be related to positive mental health among older adults.

This study first assumed that family emotional interaction may be associated with older adults' social activity. Emotional support within the family provides a sense of belonging that increases older adults' confidence and self-efficacy, which leads to a greater willingness to engage in social activities outside their homes (10). On the contrary, negative interaction leads to loneliness and social isolation, undermining older adults' confidence and motivation to participate in social activities.

Family members' attitudes toward older adults can affect older adults' self-perceptions of aging. Families that are filled with ageism regarding older adults as aged and vulnerable can trigger more negative attitudes toward aging, compelling older adults to perceive themselves as helpless, depending on others, and valueless (11). Emotional support, however, is an effective way to combat age discrimination by promoting intergenerational reciprocity and decreasing the negative consequences of social exclusion (12). Emotional support reduces the psychological burden of aging and puts older adults in a relaxed, confident state, focused on enjoying family interactions. Therefore, we argue that emotional support may promote positive attitudes toward aging among older adults.

Attitudes toward aging are inherently adaptable and can be changed by one's behavior. Informal social engagement predicted more positive perceptions of aging, and those more active in their aging status produced a positive self-fulfilling prophecy about their social performance (13). Participating in various social activities enhances positive attitudes toward aging (14). Even older adults with more negative attitudes toward aging can benefit from intervention projects that promote participation in social activities with the purpose of behavioral modification (15). Given these findings, we suggest that engagement in social activities might affect older adults' attitudes toward aging.

### 1.3 The present study

Previous research has primarily used broader social interaction structures (16, 17). The present study, on the other hand, focuses specifically on emotional interactions between family members and older adults, and how family emotional interactions create distinct psychosocial pathways that influence positive mental health. Based on the reviewed social interaction literature, we conceptualized family emotional interactions as emotional support and negative interactions in family communication. This operationalization retains the methodological advantage of measuring observable interaction behaviors.

According to self-determination theory, individuals need external social support to achieve self-integration (18). Empirical evidence indicates that emotional support from others can motivate older adults to remain socially active. Involvement in social activities has been shown to promote a more positive perception of aging.

Older adults frequently benefit from ongoing emotional support to maintain a positive perspective on the aging process. Attitudes toward aging are regarded as intrinsic psychological characteristics, which are more challenging to interfere with directly. Alterations to these attitudes require changes to occur through external factors. Attitudes toward aging are modified by external support and individual active behavior, which may, in turn, fundamentally enhance positive mental health.

The present study first aims to revise a family emotional interaction questionnaire for older adults, and second to construct an emotional support model. The second aim is to examine whether the relationship between family emotional interaction and positive mental health is mediated via social activity participation and attitudes toward aging. Specifically, it is hypothesized that emotional support will result in more social activity engagement and positive attitudes toward aging, which in turn will be positively associated with positive mental health among older adults.

## 2 Materials and methods

### 2.1 Data collection and sampling

Samples were selected using a combination of cluster and random sampling methods. Survey locations covered the communities, residential institutions, and rural villages in China's Shanxi, Shaanxi, and Henan provinces. Participants were recruited through on-site advertising and advocacy by administrators of local urban resident committees, rural village committees, and residential institutions. The recruitment criteria were older adults aged sixty and above. We conducted face-to-face interviews in participants' homes and public areas of neighborhoods, villages, and residential institutions, each lasting ~20 min.

One thousand two hundred older adults participated in this study (*M*_*age*_ = 68.49, *SD* = 7.09), including 683 females (57%). 74.3% of the participants were married, 219 participants were widowed (18.3%). The average number of years of education for the total sample was 7.26 (*SD* = 3.93). According to China's administrative division level, the locations of this survey were classified as rural areas if they were in townships, villages, and urban areas if they were in counties and cities. In this survey, there were 537 older adults living in rural areas, accounting for 44.8% of the total sample.

We first explained the purpose of the study to all participants. They signed a written informed consent form identifying their voluntary participation in the survey and allowing participants to withdraw at any time. They were thanked and rewarded after completing the questionnaires. Each respondent who completed the questionnaire was awarded 20 yuan (about US$2.80). The Ethics Committee of Xi'an Jiaotong University Ethics Review approved the survey.

### 2.2 Measures

#### 2.2.1 Family emotional interaction

We initially reviewed the research on social interactions among older adults. Lincoln et al. (6) classified the social relationship quality of older adults into two dimensions: emotional support (understanding, dependability, and open communication) and negative interactions (over-demanding, critical, and disappointing). Cheng et al. (19) proposed a positive exchange (including listening, emotional expression, and respect) vs. negative interactions (conflict, demanding, and irritating) among older adults in Hong Kong. Vella et al. (16) classified positive exchanges into four dimensions: agreeableness, intimacy, instrumental support, and emotional support. Newsom et al. (20) also distinguished positive social exchanges (e.g., affectionate behaviors) from negative social exchanges (e.g., hostility, indifference), with four entries in each dimension. Krause and Rook (17) focused on negative interaction patterns, including global negative interaction and negative interaction with children/relatives/friends and spouses (harshness, excessive expectations, controlling, etc.).

Based on previous research, there appears to be a need for questionnaire adaptation in the assessment of family interaction, as existing instruments in the literature demonstrate limitations in capturing specific aspects relevant to the current research context. A modified instrument may be beneficial in better meeting this study's unique needs.

Firstly, existing measures in social interaction research, such as Lincoln et al.'s (6) 3-item questionnaire and Cheng et al.'s (19) 4-item per dimension questionnaire, use relatively limited item pools. While these frameworks provide fundamental insights, methodological considerations suggest broadening the assessment items to represent better older adults' interpersonal experiences, including unexamined but critical behaviors (e.g., support, encouragement, and praise).

Secondly, cultural adaptation can enhance the contextual relevance of established instruments, as exemplified by Krause and Rook's (17) scale, which contains predominantly individualistic conceptualizations (e.g., emphasis on autonomy). Cross-cultural research frameworks highlight the importance of adjusting measurement instruments to the socio-cultural norms of the target population. The expressions of established instruments have limited validity in collectivist cultures. They must be adapted to linguistic or behavioral representations to fit the cultural context examined in this study.

Finally, the studies showed commonalities in the core dimensions (supportive vs. negative). Measures of interaction quality in older adults typically use the dual dimensions of emotional support and negative interaction to capture the complex effects of family emotional interactions on positive mental health.

Accordingly, this study employed a methodological synthesis of validated instruments, augmenting established measures with linguistic modifications to ensure culturally relevant operationalization aligned with our research objectives.

Family emotional interaction was assessed by asking participants to recall how they experienced when their spouse and children interacted with them. The original questionnaire contained 18 items, nine emotional support, and nine negative interaction items. The items of this questionnaire were collected primarily through a literature review, as shown in Table 1. A Likert 6-point scale was used for scoring, ranging from 1 (never) to 6 (always), with higher scores indicating more emotional support or negative interactions.

**Table 1:** Rotated factor loadings on the original family emotional interaction questionnaire.

**Item**	**Item source**	**Factor loadings**	
		**Emotional support**	**Negative interaction**
1. → Supporting your thoughts and decisions	Added	**0.747**	−0.167
2. → Encouraging you mentally	Added	**0.826**	−0.143
3. → Praising you for something.	Added	**0.803**	−0.108
4. → Understanding your feelings and opinions	Lincoln et al. (6)	**0.830**	−0.174
5. → Listening patiently to what you are saying	Cheng et al. (19)	**0.830**	−0.136
6. → Being able to speak freely and openly with them and to speak from the heart	Lincoln et al. (6)	**0.815**	−0.085
7. → Spending time with you spontaneously	Added	**0.739**	0.006
8. → Expressing gratitude to you	Cheng et al. (19)	**0.785**	−0.065
9. → Doing actions of love and care for you (e.g., hugging, speaking with attention, smiling, glancing kindly)	Vella et al. (16)	**0.675**	0.064
10. → Excessive concern for you, offering lots of advice you don't need	Newsom et al. (20)	0.227	0.213
11. → Rejecting you	Added	−0.027	**0.730**
12. → Hurtful or abusive comments about you	Added	0.014	**0.784**
13. → Criticizing you	Krause and Rook (17)	−0.066	**0.810**
14. → Disputes or quarrels with you about various things	Newsom et al. (20)	−0.052	**0.778**
15. → Excessive or overbearing demands on you	Krause and Rook (17)	−0.046	**0.811**
16. → Letting you down when you needed help or care	Newsom et al. (20)	−0.160	**0.777**
17. → Neglecting your needs and feelings	Newsom et al. (20)	−0.191	**0.715**
18. → Complaining about you for all sorts of things	Added	−0.146	**0.746**

Factor loadings over 0.40 appear in bold.

We conducted a preliminary exploratory factor analysis (EFA), and two factors were extracted using principal component analysis (PCA) with a varimax rotation. The original 18 items and their respective rotated factor loadings are shown in Table 1. Items with factor loadings equal to or >0.4 on the corresponding dimension were retained. Cronbach's alpha was 0.922 for the 9-item emotional support subscale and 0.846 for the 9-item negative interaction subscale.

However, item 10, “Excessive concern for you, offering lots of advice that you don't need,” showed that the rotated factor loadings did not belong to the emotional support or negative interaction. After removing item 10, the Cronbach's alpha for the 8-item negative interaction subscale was 0.903.

The Pearson's correlation coefficient for the total emotional support and negative interaction subscales scores was −0.220, *p* < 0.001. The fit indices using Normed Chi-square <3, RMSEA < 0.05, CFI > 0.9, TLI > 0.9, and SRMR < 0.05 suggest a good model fit (21). The two-factor model was found to best fit the hypothesis with fit indices of χ^2^= 301.469, *df* = 95, χ^2^*/df* = 3.173, RMSEA = 0.043, CFI = 0.984, TLI = 0.977, SRMR = 0.045.

The final questionnaire contained 17 items divided into two dimensions: emotional support and negative interaction. The emotional support dimension had nine items: support, encouragement, praise, understanding, listening patiently, open hearts, companionship, gratitude, and positive body and facial expressions. The negative interaction dimension had eight items: rejection, hurtful comments, criticism, arguments, excessive demands, disappointment, neglect, and complaints. The final questionnaire showed good reliability and validity.

#### 2.2.2 Social activity engagement

Participants were asked to report how often they had engaged in various social activities in the last 3 months. Using a range of activities: watching art performances, going on trips, reading, enjoying art shows, practicing calligraphy, painting, writing, gardening, volunteering, attending a senior citizen's college, playing sports, gardening, farming, taking care of family members, taking care of grandchildren, visiting relatives and friends, etc. (22). They were classified into seven categories: aesthetic activities, cognitive training activities, recreational activities, physical activities, livelihood activities, social interaction activities, and mental health promotion activities.

The engagement frequency of each activity was based on a 5-point scale: 1 (never), 2 (twice a month), 3 (once a week), 4 (2–3 times a week), and 5 (every day). The total score is the sum of the engagement frequency of older adults' social activities. A higher total score indicates a higher level of engagement. The Cronbach's alpha for the social activity engagement questionnaire was 0.90.

#### 2.2.3 Attitudes toward aging

We used the short version of the Attitudes to Aging Questionnaire with 12 items (AAQ-12) (23). It assesses older adults' attitudes toward aging on three domains: psychosocial loss, psychological growth, and physical change, with four items for each subscale. Using a five-point Likert scale, ranging from 1 (completely disagree) to 5 (completely agree). Psychosocial loss represents negative attitudes toward aging, and psychological growth and physical change represent positive attitudes toward aging. The Cronbach's alpha in the current study were 0.70, 0.64, and 0.70 for psychosocial loss, psychological growth, and physical change, respectively.

#### 2.2.4 Positive mental health

To measure positive mental health, we used the Chinese version of the Mental Health Continuum Short Form (MHC-SF) (24). The scale consisted of 14 items, including three subscales: emotional wellbeing (EWB), psychological wellbeing (PWB), and social wellbeing (SWB). We calculated the total score of all items and each subscale using a six-point Likert scale, ranging from 0 (never) to 5 (every day). Higher scores indicate higher positive mental health. Cronbach's alpha in the current study were 0.86, 0.81, 0.70, and 0.80 for the MHC-SF, EWB, PWB, and SWB, respectively.

### 2.3 Data analysis

SPSS 27.0 was used for primary analysis. Descriptive statistics (means, standard deviations, frequencies, and percentages) were used to describe the study sample and the main variables: family emotional interaction, social activity engagement, attitudes toward aging, and positive mental health. Pearson's correlation was used to examine the correlations between the main variables. Amos 24.0 was used to test the hypothesized mediating model of family emotional interaction and positive mental health after controlling for gender and residency. A bias-corrected percentile bootstrap method was used to examine the mediating effects. Bootstrap 95% confidence intervals (95% CI) for mediating effects were calculated by repeated random sampling of 5,000 Bootstrap samples from the original data. None of the 95% confidence intervals for the mediated paths included 0, indicating they were all statistically significant (25). Model fit indices were a Chi-square value divided by a degree of freedom (χ^2^*/df*) < 5, a comparative fit index (CFI) and a Tucker-Lewis Index (TLI) > 0.90, a root mean square error of approximation (RMSEA) value and standardized root mean square residual value (SRMR) < 0.05 indicated excellent fit to the data (26, 27).

## 3 Results

### 3.1 Descriptive and inferential analyses

Table 2 shows the descriptive statistics and Pearson correlation between the study variables. A significant correlation emerged between negative interaction, positive mental health, and attitudes toward aging, except with social activity engagement. Furthermore, psychosocial loss was negatively correlated with social activity engagement, emotional support, and positive mental health.

**Table 2:** Descriptive statistics and Pearson correlation between the main variables.

**Variables**	**1**	**2**	**3**	**4**	**5**	**6**	**7**	**8**	**9**	**10**
1. Emotional support	-									
2. Negative interaction	−0.126^**^	-								
3. Social activity engagement	0.330^**^	0.031	-							
4. Psychological growth	0.316^**^	−0.159^**^	0.344^**^	-						
5. Psychosocial loss	−0.106^**^	0.195^**^	−0.153^**^	−0.149^**^	-					
6. Physical change	0.249^**^	−0.219^**^	0.341^**^	0.496^**^	−0.245^**^	-				
7. EWB	0.259^**^	−0.223^**^	0.203^**^	0.248^**^	−0.178^**^	0.304^**^	-			
8. SWB	0.242^**^	−0.176^**^	0.331^**^	0.235^**^	−0.149^**^	0.295^**^	0.456^**^	-		
9. PWB	0.331^**^	−0.108^**^	0.416^**^	0.384^**^	−0.130^**^	0.380^**^	0.507^**^	0.595^**^	-	
10. PMH	0.341^**^	−0.187^**^	0.406^**^	0.364^**^	−0.175^**^	0.401^**^	0.721^**^	0.827^**^	0.904^**^	-
M	4.048	1.982	2.534	3.476	2.597	3.711	3.620	3.246	3.247	3.327
SD	1.006	0.821	0.641	0.878	0.993	0.861	1.125	0.940	1.104	0.879

^**^ Means *p* < 0.01.

### 3.2 Model analysis

**Figure 1:**
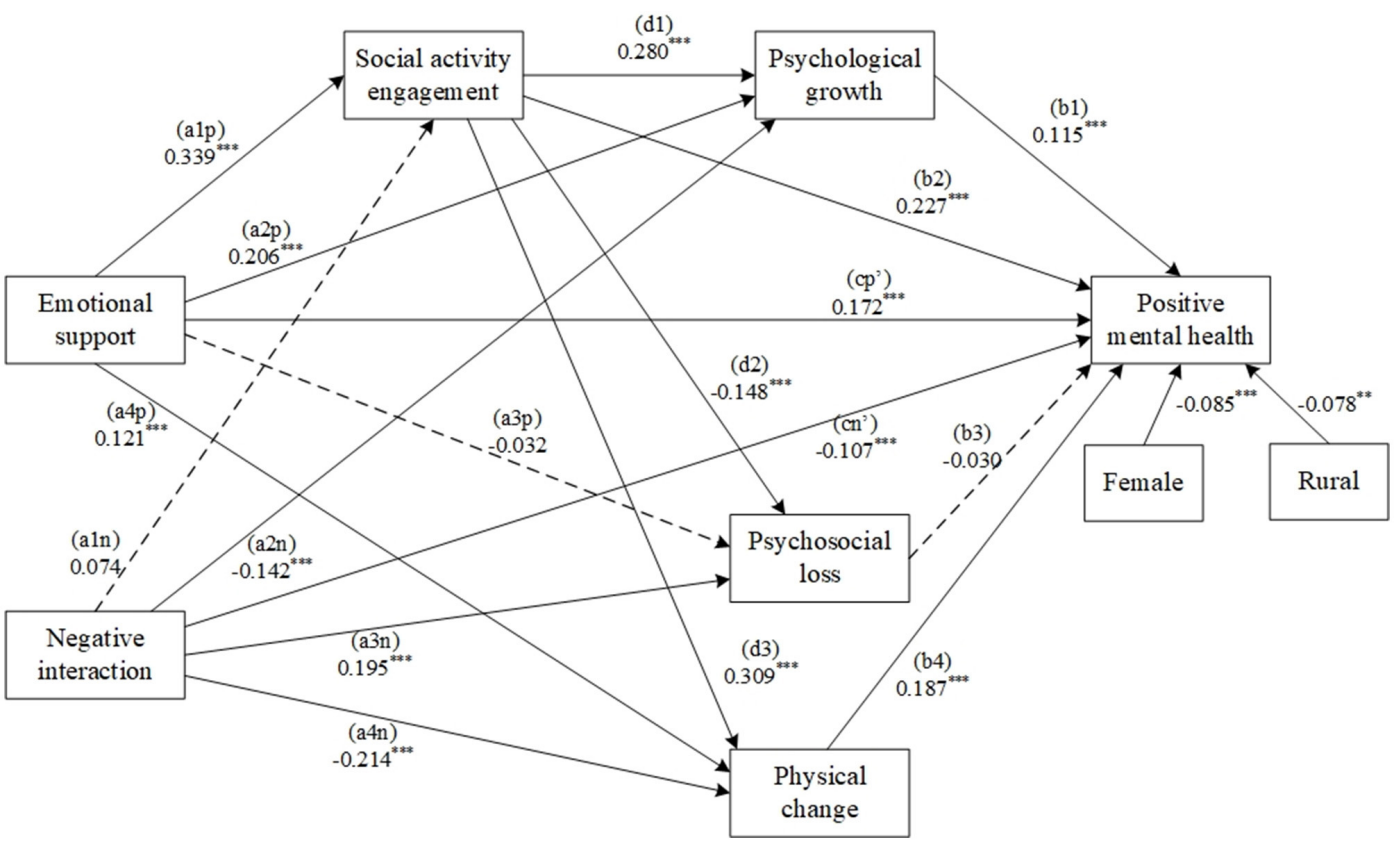
An emotional support model with social activity engagement and attitudes toward aging as the chain mediators. The standardized regression coefficients are outside parentheses. Non-significant paths are marked using dashed lines. *** Means *p* < 0.001.

To test the main hypothesis: social activity engagement and attitudes toward aging chain mediated the association between emotional support and positive mental health among older adults. We built a model that included all paths of emotional support, social activity engagement, attitudes toward aging, and positive mental health after controlling for gender and residence. Emotional support and negative interactions were placed in the same model as a comparison (Figure 1). The goodness-of-fit indicators of the emotional support model were χ^2^*/df* = 2.466, TLI = 0.966, CFI = 0.992, RMSEA = 0.035, and SRMR = 0.019.

As Table 3 shows, the paths between emotional support, social activity engagement, positive attitudes toward aging (psychological growth and physical change), and positive mental health were all statistically significant (*ps* < 0.001). The paths between negative interaction, positive attitudes toward aging (psychological growth and physical change), and positive mental health were all statistically significant (*ps* < 0.001). Statistical analyses revealed non-significant associations between emotional support and psychosocial loss, as well as between negative interactions and social activity engagement. Consequently, neither the psychosocial loss mediation pathway nor the serial mediation pathway involving negative interactions and social activity engagement demonstrated statistical viability.

**Table 3:** The emotional support model path analysis with social activity engagement and attitudes toward aging as mediators.

**Labels**	**Paths**	**B**	**β**	***p***
a1p	Emotional support → Social activity engagement	0.696	0.339	<0.001
a2p	Emotional support → Psychological growth	0.080	0.206	<0.001
a3p	Emotional support → Psychosocial loss	−0.014	−0.032	0.281
a4p	Emotional support → Physical change	0.046	0.121	<0.001
a1n	Negative interaction → Social activity engagement	0.209	0.074	0.107
a2n	Negative interaction → Psychological growth	−0.076	−0.142	<0.001
a3n	Negative interaction → Psychosocial loss	0.118	0.195	<0.001
a4n	Negative interaction → Physical change	−0.112	−0.214	<0.001
b1	Psychological growth → PMH	0.403	0.115	<0.001
b2	Social activity engagement → PMH	0.151	0.227	<0.001
b3	Psychosocial loss → PMH	−0.093	−0.030	0.232
b4	Physical change → PMH	0.673	0.187	<0.001
d1	Social activity engagement → Psychological growth	0.053	0.280	<0.001
d2	Social activity engagement → Psychosocial loss	−0.032	−0.148	<0.001
d3	Social activity engagement → Physical change	0.057	0.309	<0.001
cp	Emotional support → PMH	0.397	0.292	<0.001
cp'	Emotional support → PMH	0.234	0.172	<0.001
cn	Negative interaction → PMH	−0.252	−0.135	<0.001
cn'	Negative interaction → PMH	−0.200	−0.107	<0.001

Table 4 shows the results of the mediation analysis. We identified four mediating paths: Path 1 Social activity engagement and psychological growth chain mediated the association between emotional support and positive mental health, with an effect size of 0.015, 95% CI [0.007, 0.025]. Path 2 Social activity engagement and physical change chain mediated the association between emotional support and positive mental health, with an effect size of 0.027, 95% CI [0.017, 0.040]. Path 3 Psychological growth mediated the association between negative interaction and positive mental health, with an effect size of −0.031, 95% CI [−0.053, −0.015]. Path 4 Physical change mediated the association between negative interaction and positive mental health, with an effect size of −0.075, 95% CI [−0.110, −0.048].

**Table 4:** The mediate effects and total effects of the emotional support model.

**Labels**	**Paths**	**Effect size**	**95%CI**	**% of total effect**
a1 * b2	Emotional support → Social activity engagement → PMH	0.105	[0.074, 0.414]	27.2%
a2 * b1	Emotional support → Psychological growth → PMH	0.032	[0.016, 0.052]	8.3%
a1p * d1 * b1	Emotional support → Social activity engagement → Psychological growth → PMH	0.015	[0.007, 0.025]	3.9%
Total 1		0.386	[0.302,0.476]	
a4 * b4	Emotional support → Physical change → PMH	0.031	[0.015, 0.052]	7.8%
a1 * d3 * b4	Emotional support → Social activity engagement → Physical change → PMH	0.027	[0.017, 0.040]	6.8%
Total 2		0.397	[0.309, 0.480]	
a2n * b1	Negative interaction → Psychological growth → PMH	−0.031	[−0.053, −0.015]	13.4%
Total 3		−0.231	[−0.324, −0.135]	
a4n * b4	Negative interaction → Physical change → PMH	−0.075	[−0.110, −0.048]	27.3%
Total 4		−0.275	[−0.371, −0.182]	

## 4 Discussion

This study was conducted to investigate the mediating effects of social activity engagement and attitudes toward aging on the relationship between family emotional interaction and positive mental health. From the multidimensional perspective of family emotional interaction, emotional support directly correlates with positive mental health, while simultaneously exerting indirect effects through enhanced social activity engagement and improved attitudes toward aging. This dual-path mechanism constitutes the conceptual framework of the emotional support model proposed in this study, whereby relational provisions interact with behavioral and cognitive mediators to optimize positive mental health among older adults.

We first revised a family emotional interaction questionnaire for older adults based on existing research. The final questionnaire included 17 family emotional interaction behaviors and demonstrated satisfactory psychometric properties. In contrast to previous studies, our study examined the quality of older adults' family interactions by including a wide range of items that fully reflect the behaviors that may occur in daily interactions with their family members.

The results supported the hypothesized emotional support model. Within the model, emotional support is the main effect of promoting positive mental health among older adults. It contributes to older adults' increased participation in social activities and the development of positive aging attitudes, resulting in higher positive mental health. It implies that emotional support may be essential in improving older adults' overall wellbeing.

The stress-buffering model proposes two crucial emotional supports: esteem support and social companionship (28). Positive family interaction provides adequate self-esteem support and social companionship, thus constructing a support system beneficial to an individual's sense of security. Previous research has found that older adults living in supportive environments are significantly more likely to participate in activities (29). Conversely, the absence of emotional support is a significant barrier to regular exercise and physical activity among older adults (30).

According to self-perception theory, people infer or change their attitudes and emotions by observing their behavior (31). Intervention studies found that social activity directly affected an individual's self-perception, as the more individuals participated in social activities during the interventions, the more significant the improvement in strength and physical self-perceptions (32). Older adults with higher activity engagement were more likely to perceive themselves as energetic and valued and less likely to develop negative age stereotypes, enhancing positive mental health.

Our study also found that positive attitudes toward aging mediated the association between negative interaction and positive mental health among older adults. It suggests that negative interaction as a source of stress decreases older adults' positive aging attitudes, lowering their positive mental health. Negative family interaction and fragile family relationships can make it difficult for older adults to feel a sense of belonging, and they tend to perceive themselves as unwanted or expendable and as burdens to the family (33). Such a thwarted sense of belonging and burdensomeness can harm an individual's wellbeing (34). Our results further emphasize the importance of emotional support in promoting positive mental health among older adults.

The emotional support model of our study aligns with the principles of Self-Determination Theory (SDT). Emotional support has been shown to enhance positive mental health by increasing older adults' intrinsic motivation to engage in social activities autonomously, thereby improving their sense of worth (e.g., positive attitudes toward aging) and satisfying relational needs (e.g., a sense of belonging to the family). The differential impact of emotional support and negative interactions underscores the dual process nature of family emotional interactions, whereby supportive behaviors and harmful exchanges work through different psychosocial pathways.

## 5 Limitation

This study had some limitations. First, for the final version of the family emotional interaction questionnaire, we eliminated the item of excessive concern. The excessive concern item originated from Newsom et al. (20) and was regarded as a negative interaction. However, our study found that excessive concern for older adults is neither a negative interaction nor emotional support in the Chinese socio-cultural context. Older adults perceived family members' excessive concern as feeling bothered and cared for simultaneously, which is a more complex emotion. Future studies consider further analyzing and investigating such complex interaction dimensions. Second, as a cross-sectional study, there are limits to examining the causal links between family interactions, social activity, aging attitudes, and positive mental health. Still, our results are consistent with previous studies that utilized longitudinal data. Finally, a potential limitation of the study is the possibility of unavoidable sample selection bias, despite using both cluster and random sampling methods and controlling demographic variables. Future studies may employ stratified random sampling to enhance the samples' representativeness and expand the sampling frame and sample size.

## 6 Conclusion

This study presented an emotional support model among older adults: social activity engagement and positive attitudes toward aging mediated the association between emotional support and positive mental health. Being emotionally supported and actively participating in social activities are essential strategies for older adults to cope with potential stressors in later life. These findings highlight the necessity for incorporating family-centered interventions within the gerontological practice, particularly through evidence-based approaches such as systemic family therapy and multigenerational support programs, to optimize emotional support across the aging trajectory.

## Data Availability

The raw data supporting the conclusions of this article will be made available by the authors, without undue reservation.
